# Detection of T lymphocyte subsets and related functional molecules in follicular fluid of patients with polycystic ovary syndrome

**DOI:** 10.1038/s41598-019-42631-x

**Published:** 2019-04-15

**Authors:** Zitao Li, Anping Peng, Yuanfa Feng, Xiaona Zhang, Fenghua Liu, Chuangqi Chen, Xin Ye, Jiale Qu, Chenxi Jin, Mei Wang, Huaina Qiu, Yanwei Qi, Jun Huang, Quan Yang

**Affiliations:** 1 Reproductive medical center, Guangdong Women and Children Hospital, Guangzhou Medical University, 511400 Guangzhou, China; 2 Clinical laboratory, Traditional Chinese Medicine Hospital of Guangdong province, 510120 Guangzhou, China; 3 Department of Pathogenic Biology and Immunology, Sino-French Hoffmann Institute, Guangzhou Medical University, 511436 Guangzhou, China; 4 The Sixth Affiliated Hospital of Sun Yat-Sen University, 510655 Guangzhou, China; 5 State Key Laboratory of Respiratory Disease, Guangzhou Institute of Respiratory Health, The First Affiliated Hospital of Guangzhou Medical University, 510120 Guangzhou, China

## Abstract

Immune responses play an important role in the pathogenesis of polycystic ovary syndrome (PCOS). However, the characteristics of T lymphocyte subsets in PCOS remain insufficiently understood. In this study, lymphocytes of follicular fluid (FF) were obtained from oocyte retrieval before *in-vitro* fertilization (IVF) in infertile women with or without PCOS. The levels of cluster of differentiation 25 (CD25), CD69, programmed death 1 (PD-1), interferon-γ (IFN-γ), interleukin 17A (IL-17A) and IL-10 in T lymphocytes were determined by flow cytometry. Our results showed that the percentage of FF CD8^+^ T cells was significantly decreased in infertile patients with PCOS (*P* < 0.05). Furthermore, the levels of CD69 and IFN-γ were significantly decreased and the level of PD-1 was increased in both CD4^+^ and CD8^+^ T cells from infertile patients with PCOS (*P* < 0.05). Moreover, the expression of PD-1 on CD4^+^ or CD8^+^ T cells was positively correlated with the estradiol (E2) levels in the serum and reversely correlated with the expression of IFN-γ in CD4^+^ or CD8^+^ T cells in infertile patients with PCOS. These results suggested that T cell dysfunction may be involved in the pathogenesis of PCOS.

## Introduction

Polycystic ovary syndrome (PCOS), as a common female endocrinopathy at reproductive age, is a heterogeneous condition characterized by clinical symptoms, including reproductive, cardiometabolic, and psychological disorders^1,2^. Patients with PCOS are at a significantly higher risk for the development of endometrial, breast and ovarian cancers and symptomatic atherosclerotic cardiovascular diseases (CVD)^3,4^. Other manifestations include hyperinsulinism, insulin resistance, obesity, diabetes, hirsutism, endothelial dysfunction, and a state of low-grade inflammation^5–9^. Besides, recent study have reported that insulin resistance, compensatory hyperinsulinemia and increased androgen production have potential effected on the pathogenesis of PCOS^10,11^. Although previous studies show that both environmental and genetic factors play roles in the etiology of PCOS^12,13^, the pathogenesis of PCOS is not fully understood.

The immune system is a defense system, which comprises many biological structures that protect the host against disease. Once the body’s immune system is dysfunctional, it can lead to various diseases. A recent study has reported that immunological mechanisms are involved in the regulation of polycystic ovary syndrome^14^. Patients with PCOS have been found to be under a chronic low-grade inflammation status, including high levels of leukocytes, endothelial dysfunction, and disorder of the proinflammatory cytokines^15–17^. Large amounts of immunocompetent cells, including T cells, B cells, macrophages and dendritic cells, have been found in human preovulatory follicles^18–20^. As the main component of lymphocytes, T cells have various biological functions, which are mainly involved in the cellular immune response of the body. They can kill target cells directly or through the release of lymphatic factor to enhance and expand the immune effect^21,22^. According to different functions, T cells can be classified into three subtypes: helper T cells (CD3^+^CD4^+^ Th) and cytotoxic T cells (CD3^+^CD8^+^ Tc). CD4^+^ T helper cells can be subdivided into different subsets, including Th17 cells, which produce IL-17A, and IL-17F, Th1 cells, which produce IFN-γ, IL-2, and TNF-α, Th2 cells, which secrete IL-4, IL-5, and IL-13, and Regulatory T cells, which express Foxp3, IL-10, and TGF-β^23^. Although T cells have been reported to exist in pre-ovulation follicles in humans and the interaction between subtypes is very active^24,25^, the role of T lymphocyte subsets in the pathogenesis of PCOS remains unclear.

Although substantial evidence has indicated that inflammation, as well as immune regulation might play important roles in the cause of PCOS, the underlying regulatory mechanisms have remained unclear^14,15^. The aim of this study is to investigate the subpopulations and related functional molecules of T lymphocytes in the FF of infertile women with or without PCOS. Our results will provide a better understanding of the immunoregulatory mechanism in the pathogenesis of PCOS.

## Methods

### Ethics statement

The research followed the tenets of the Declaration of Helsinki. Informed consents were obtained from all patients. And all the enrolled patients participated in the research voluntarily and freely. Our research were approved by the Institutional Review Board (IRB approval number: 201701042) of the Guangdong Women and Children Hospital. Our study conformed to the international guidelines available through the Enhancing the QUAlity and Transparency Of health Research (EQUATOR) network.

### Patient characteristics

Sixty-six primary infertile women undergoing *in vitro* fertilization (IVF) or intracytoplasmic sperm injection (ICSI) were enrolled in the study (age range: 23–37 years). Among all patients, 36 cases enrolled in the study were normally ovulating women (NOW, group A) and 30 cases were affected by PCOS (group B). Patients affected by other significant gynecological and non-gynecological comorbidities were excluded. Before admission to the study, each woman underwent clinical and transvaginal ultrasonography. Basic sexual hormones, including estradiol (E2), androstenedione (A), progesterone (P), testosterone (T), cortisol, luteinizing hormone (LH) and follicle stimulating hormone (FSH), were evaluated. The characteristics of all patients are summarized in Table 1.

**Table 1:** Comparison of related indicators for two groups.

	group B (PCOS, n = 30)	group A (NOW, n = 36)	t value	p value
Age (year)	30.30 ± 6.23	29.50 ± 4.22	0.345	0.731
FSH (IU/L)	6.15 ± 1.95	6.92 ± 1.93	−1.297	0.199
LH (mIU/L)	10.78 ± 5.91	5.54 ± 3.65	3.049	0.008
LH/FSH	1.75 ± 0.69	0.85 ± 0.58	4.826	<0.001
E2 (pg/L)	58.70 ± 52.75	53.57 ± 56.06	0.299	0.766
T (ng/mL)	0.61 ± 0.48	1.08 ± 4.95	−0.341	0.734

*p-values reported are the results of independent-sample *t*-tests or γ^2^ tests for dichotomous variables; x ± s; *p < 0.05. PCOS: polycystic ovary syndrome; NOW: normally ovulating women; FSH: follicle stimulating hormone; LH: luteinizing hormone; E2: estradiol; T: testosterone.

The control group and PCOS group were in line with the normal distribution tested with SPSS. The inclusion criteria in group A were: the absence of endocrinological disorders of the pituitary or ovary, such as hyperprolactinemia, hypogonadotropic hypogonadism, premature ovarian failure and premature menopause, or of abnormal adrenal or thyroid function. Previously reported criteria for PCOS were employed^26–28^, which include at least two of the following three criteria: 1. Oligo- and/or anovulation; 2. Clinical and/or biochemical signs of hyperandrogenism; or 3. Polycystic ovaries (presence of 12 or more follicles in each ovary measuring 2 ± 9 mm in diameter and/or increased ovarian volume), as well as the exclusion of nonclassic congenital adrenal hyperplasia, Cushing’s Syndrome, hyperprolactinemia and thyroid diseases.

### Sample Size

In our study, a sample size of 30 cases in PCOS group and 36 cases in NOW group with infertility was obtained from the two groups whose T cell subsets frequencies were compared. We performed a sample size calculation according to two independent design data calculation formulas post-hoc. We calculated the sample size according to the difference between the two groups of T cells and the power of this study was 0.8. The result we got was that each group needed 35 cases. We tried our best to collect the total number of cases close to our expected sample size. Due to the large number of testing items, we have collected relatively few complete testing cases. However, the data were true and reliable. Owing to the small sample size of our study, we could not exclude that a type 1 error might occur in our statistical analysis.

### Controlled ovarian hyperstimulation (COH)

In PCOS patients with infertility, rFSH [Gonal-F alfa (Merck Serono, Geneva, Switzerland) or Puregon beta (MSD, New Jersey, USA)] treatment was initiated on menstrual cycle day 2 or day 3. The starting doses were 112.5–300 IU per day selected based on the age, circulating basal FSH level and BMI of patients. The rFSH doses were adjusted according to growing follicles and E2 concentration during the stimulation monitoring. The GnRH antagonist treatment (Ganirelix 0.25 mg, Orgalutran®, Organon, Italy) was initiated on stimulation day 5–7 as the growing follicles 10–12 mm in diameter.When at least three dominant follicles (diameter ≥17 mm) were observed by ultrasound, 250 μg rhCG (Choriogonadotropin alfa, Merck Serono, Geneva, Switzerland) was administered to trigger the final oocyte maturation. Oocyte retrieval was performed 34–36 hour after rhCG administration. One or two embryos were transferred 3–5 days after oocyte retrieval. Luteal phase progesterone support (Progesterone Vaginal Gel, Merck Serono, Geneva, Switzerland) was provided.

### Follicular fluid aspiration

Individual follicles were measured before aspiration in two dimensions. When the leading follicle was calculated greater than or equal to 17 mm in diameter, 250 μg of human chorionic gonadotropin (hCG) (Merck Serono, Geneva, Switzerland) were administered to induce ovulation. Transvaginal follicular aspiration was scheduled after 36 h and performed under general anesthesia. The follicles were aspirated with a 16-gauge single lumen needle, and each follicle was emptied completely. The follicular fluid (FF) of the leading follicle was collected into a dry tube (without medium) (BD Falcon #352057; BD Biosciences, Boston, MA). In the laboratory, the cumulus oophorus complex was isolated, and the follicular fluid was subsequently centrifuged at 600 × g. The pellet was resuspended in 1 ml of RPMI 1640 medium (Invitrogen Life Technologies, Grand Island, NY, USA) and placed into a tube for further experiments. The experiments were performed within 6 hours.

### Cell surface staining

This experiment was performed following previously described procedures^29^. Single-cell suspensions from the follicular fluid of infertile women with and without PCOS were adjusted to 0.3 × 10^6^/ml, washed twice in phosphate-buffered saline (PBS) (Invitrogen Life Technologies, Grand Island, NY, USA) and blocked in PBS buffer that contained 1% bovine serum albumin (BSA) (Sigma-Aldrich, St. Louis, MO, USA) for 30 min. The cells were then stained for 30 min at 4 °C in the dark with conjugated antibodies specific for the following cell surface antigens: anti-CD3 PerCP, anti-CD4 FITC, anti-CD8 PE, anti-CD25 PE-CY7, anti-CD69 APC and anti-PD-1 Brilliant Violet 421 (eBioscience, San Diego, CA, USA). The phenotypic characteristics of the antibody-labeled lymphocytes were analyzed using flow cytometry (Beckman Coulter, Fullerton, CA, USA), and the results were analyzed using FlowJo version 6.0 software (TreeStar Inc., Ashland, OR, USA). Isotype-matched controls were included in each staining protocol.

### Intracellular cytokine staining

Previously described procedures were employed^30^. Cells (1 × 10^6^/ml) from the follicular fluid of infertile women with and without PCOS were stimulated with propylene glycol monomethyl acetate (PMA) (at a final concentration of 20 ng/ml, Sigma-Aldrich, St. Louis, MO, USA) plus ionomycin (at a final concentration of 1 μg/ml, Sigma-Aldrich, St. Louis, MO, USA) for 5 hours at 37 °C under a 5% CO_2_ atmosphere. Brefeldin A (a final concentration of 10 μg/ml, Sigma-Aldrich, St. Louis, MO, USA) was added during the last 4 hours of incubation. The cells were washed twice in PBS and then stained for 30 min at 4 °C in the dark with conjugated antibodies specific for cell surface antigens: anti-CD3 PerCP, anti-CD4 FITC, anti-CD4 PE-cy5, anti-CD8 PE, and anti-CD8 FITC (eBioscience, San Diego, CA, USA). The cells were washed twice in PBS again, fixed with 4% paraformaldehyde and permeabilized overnight at 4 °C in PBS buffer that contained 0.1% saponin (Sigma-Aldrich, St. Louis, MO, USA), 0.1% BSA and 0.05% NaN_3_ (Sigma-Aldrich, St. Louis, MO, USA). The cells were then stained for 30 min at 4 °C in the dark with conjugated antibodies specific for cytokines: anti-IFN-γ APC, anti-IL-10 PE-cy7, anti-IL-4 PE, and anti-IL-17A APC-CY7 (eBioscience, San Diego, CA, USA). The expressions of cytokines secreted by antibody-labeled lymphocytes were analyzed using a FACS, and the results were analyzed using FlowJo version 6.0 software. Isotype-matched controls for cytokines were included in each staining protocol. For the staining of Foxp3, the Foxp3/Transcription Factor Staining Buffer Set and the conjugated antibodies specific for Foxp3, anti-Foxp3 APC (eBioscience, San Diego, CA, USA), were used. After staining, the cells were washed and resuspended in PBS for flow cytometric analysis using a FACS. The data were then analyzed using FlowJo version 6.0 software.

### Cytometric bead array (CBA)

Previously described procedures were employed^17^. Cells were isolated from the follicular fluid of infertile patients with PCOS or NOW and resuspended in 200 μl of RPMI 1640 medium. The cells were then stimulated with 20 ng/ml PMA and 1 μg/ml ionomycin and incubated for 48 h. The levels of IFN-γ, IL-10, IL-4 and IL-17A in the cell culture supernatants were analyzed using a cytometric bead array kit (CBA) (Human Th1/Th2/Th17 Cytokine Kit, Becton Dickinson, San Jose, CA). Briefly, the supernatants were harvested and stored at −80 °C until cytokine determination. Then, 50 μl of each sample was mixed with 50 μl of mixed capture beads and 50 μl of the human Th1/Th2/Th17 PE detection reagent that consisted of PE-conjugated anti-human cytokines. The samples were incubated at room temperature for 3 h in the dark. After incubation with the PE detection reagent, the samples were washed once and resuspended in 300 μl of washing buffer before acquisition on a FACSCalibur cytometer (BD Biosciences). Data were analyzed using CBA software (BD Biosciences). Standard curves were generated for each cytokine using the cytokine standard provided by the kit. The concentration of each cytokine in the cell supernatant was determined by interpolation to the corresponding standard curve. The assay sensitivity is denoted by 3.7 pg/ml for IFN-γ, 4.5 pg/ml for IL-10, 4.9 pg/ml for IL-4 and 18.9 pg/ml for IL-17A. We refrained from adjusting the cell number prior to the *in vitro* stimulation because we aimed to obtain the net production of cytokines for each individual.

### Statistics

Statistical analyses of the differences between means were performed using unpaired, two-tailed tests. If the data is non-normally distributed, we used a nonparametric test to compare the difference. Statistical tests were performed using GraphPad Prism version 5.0 and SPSS Statistics 17.0. P-values of <0.05 were considered significant.

## Results

### Characteristics of infertile patients with PCOS

Before oocyte retrieval, a general clinical examination was performed. The plasma hormones and biochemical indicators were determined, including the baseline levels of T, E2, LH and FSH. Moreover, the ratio of LH to FSH was calculated. The results of the study showed that there were no significant differences in age, infertility years, or levels of T or FSH between the infertile patients with PCOS and the controls (Table 1, *P* > 0.05). However, the levels of LH and LH/FSH ratio were significantly increased in group B (Table 1, *P* < 0.01). Our data showed disparities compared with previous reports^17^. This discrepancy might be the result of the hereditary and demographic differences between Asian and European or American individuals.

### Percentages of T lymphocyte subsets in follicular fluid of infertile women with and without PCOS

**Figure 1:**
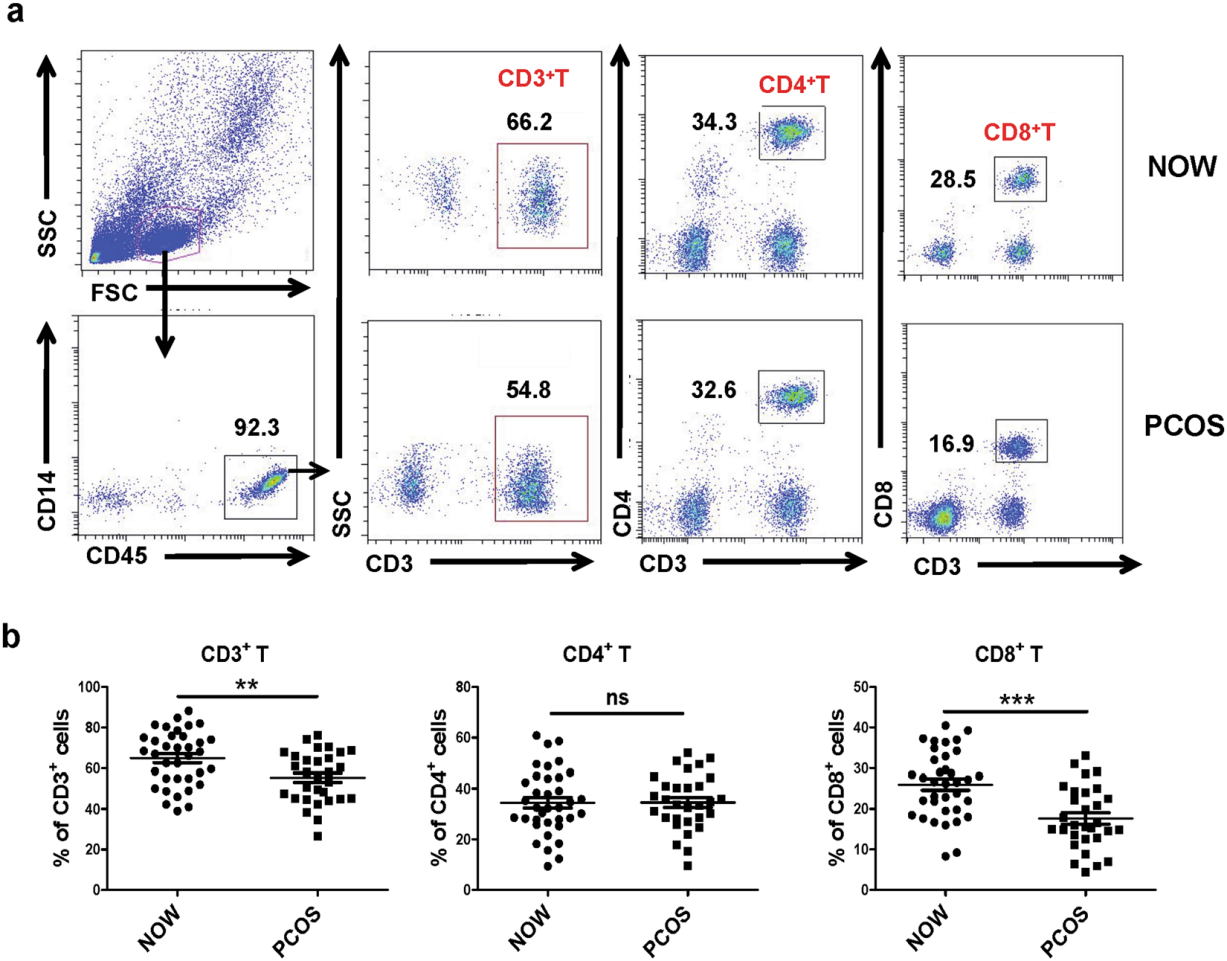
Percentages of T lymphocyte subsets in follicular fluid of infertile women with and without PCOS. Follicular fluid samples were collected from normally ovulating infertile women (NOW, n = 36) and infertile patients with PCOS (n = 30). CD45^+^CD14^−^ lymphocyte cells were first gated, and the levels of T lymphocytes and subsets were determined by flow cytometry. Representative results (**a**) and mean ± s.e.m. (**b**) are shown. **P < 0.05, ***P < 0.01, no significant difference (ns) was *P* > 0.05 compared with control group.

To observe the changes in the T lymphocyte subsets between group A and group B, lymphocytes were isolated from the follicular fluid. The cells were quantified, and the expressions of CD14, CD45, CD3, CD4 and CD25 were subsequently detected by flow cytometry. Anti-CD14 and anti-CD45 antibodies were used to confirm the population of lymphocytes (CD45^+^CD14^−^ cells). The flow cytometric analysis showed that the percentages of CD3^+^ and CD8^+^ (CD3^+^CD8^+^) T lymphocytes were significantly reduced in the follicular fluid of the infertile women with PCOS compared with the infertile women with normal ovulation (66.2% ± 2.1% vs. 54.8% ± 2.8%, P < 0.01; 28.4% ± 1.2% vs. 16.8% ± 1.4%, P < 0.01). However, the differences in the relative percentages of CD4^+^ (CD3^+^CD4^+^) between the PCOS and control group were not robust (Fig. 1a,b).

### Expression of CD25 and CD69 on the surface of CD4^+^ and CD8^+^ T cells

**Figure 2:**
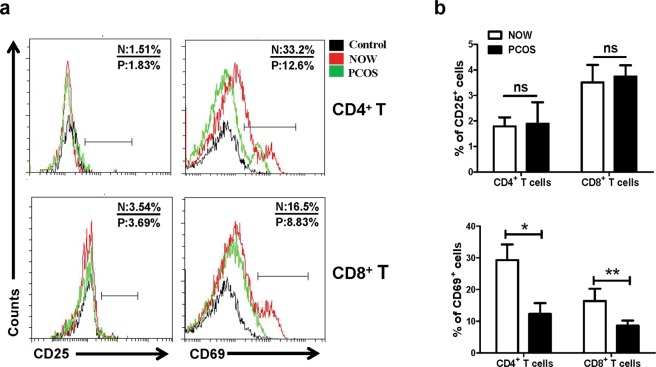
Activated CD4^+^ and CD8^+^ T cells in follicular fluid of infertile women with and without PCOS. Single cell suspensions were isolated from follicular fluid of NOW (n = 36) and patients with PCOS (n = 30). The expressions of CD25 and CD69 on T lymphocyte subsets were detected using cell surface staining, as previously described in the “Materials and methods”. The expressions of CD25 and CD69 were analyzed using flow cytometry. A representative result (**a**) and mean expressions of CD25 and CD69 were calculated from FACS data (**b**). *P < 0.05, **P < 0.01, compared with control group.

To further explore the activation state of the T lymphocyte subsets, the expressions of the activated molecules CD25 and CD69 were measured by cell surface staining. CD3^+^CD4^+^ cells and CD3^+^CD8^+^ cells were first gated, and the percentages of CD25 and CD69 on these cell populations were subsequently analyzed. As shown in Fig. 2a,b, there was no difference in the expression of CD25 on CD4^+^ or CD8^+^ T cells between the PCOS and NOW (*P* > 0.05); however, the expressions of CD69 in the PCOS group with infertility were significantly decreased both on CD4^+^ T cells (*P* < 0.05) and CD8^+^ T cells (*P* < 0.01) compared to the infertile patients with normal ovulation.

### Expressions of IFN-γ, IL-17, IL-10, IL-4 and PD-1 by CD4^+^ and CD8^+^ T cells

**Figure 3:**
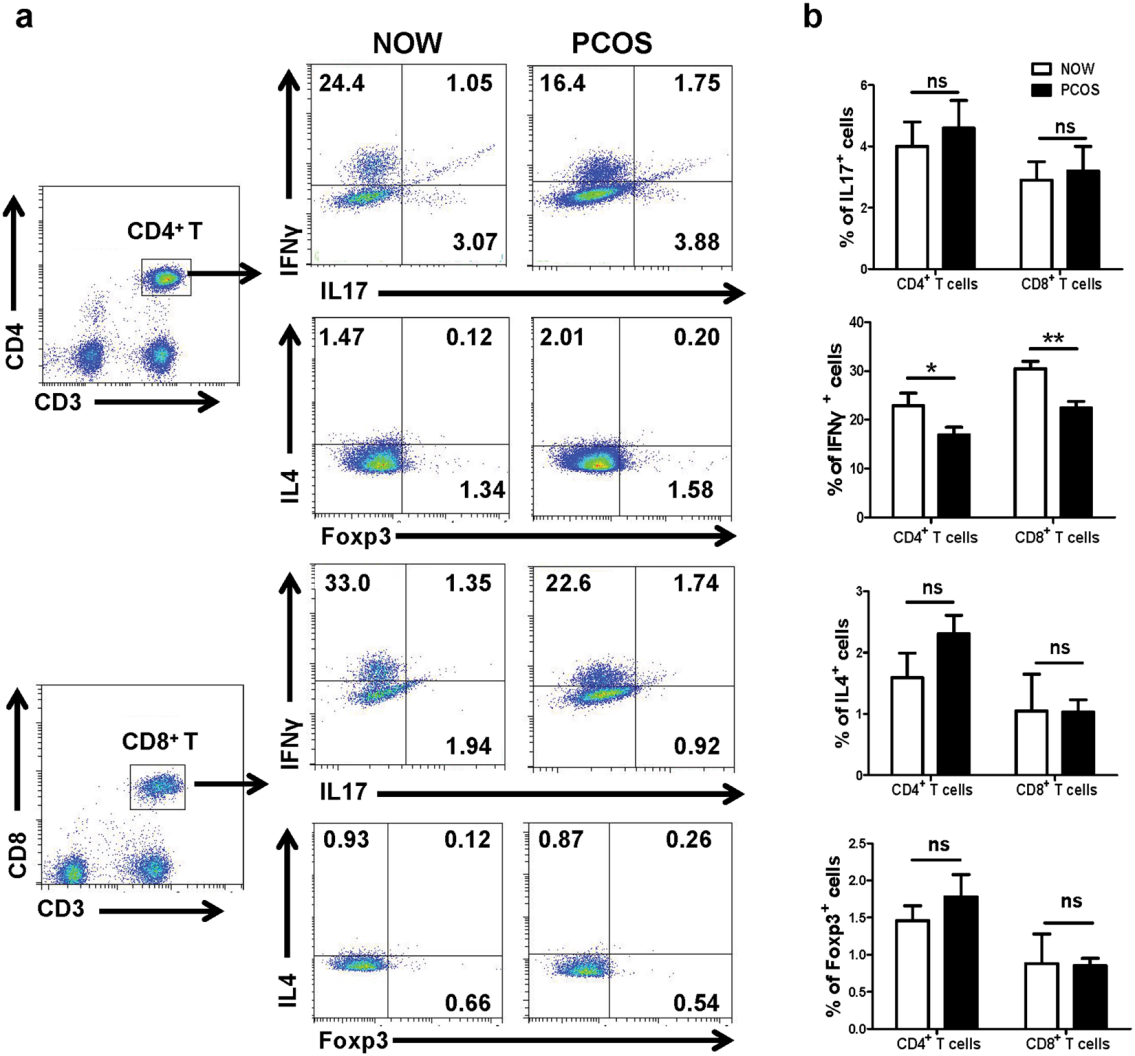
IFN-γ, IL-4, L-17A and Foxp3 expressed by CD4^+^ and CD8^+^ T cells. Cytokine expression profiles of CD4^+^ and CD8^+^ T cells from follicular fluid of NOW (n = 20) and patients with PCOS (n = 20) were determined. Single cell suspensions were stimulated with PMA plus ionomycin. CD3^+^CD4^+^ cells and CD3^+^CD8^+^ cells were first gated, and the expressions of IFN-γ, IL-4, L-17A and Foxp3 by CD4^+^ and CD8^+^ T cells were examined using intracellular cytokine or nuclear protein staining. A representative result (**a**) and mean ± s.e.m. (**b**) are shown. *P < 0.05, **P < 0.01, compared with control group.

As the percentage and activation state of the T lymphocyte subsets were different, we investigated the cytokine production of CD4^+^ and CD8^+^ T cells. Lymphocytes from the follicular fluid were isolated and adjusted to 1 × 10^6^/ml. After stimulation by PMA and ionomycin, intracellular cytokines were stained. CD3^+^CD4^+^ cells and CD3^+^CD8^+^ cells were first gated, and the results indicated that the percentages of IFN-γ-expressed CD4^+^ and CD8^+^ T cells in the infertile patients with PCOS were significantly lower than those in the infertile patients with normal ovulation (23.6% ± 3.4% vs. 16.9% ± 2.6%, *P* < 0.05; 31.8% ± 2.5% vs. 22.5% ± 2.2%, *P* < 0.01). However, no changes were identified in the percentage of IL-17 and IL-4 expressed CD4^+^ or CD8^+^ T cells between the two groups (*P* > 0.05) (Fig. 3a,b).

**Figure 4:**
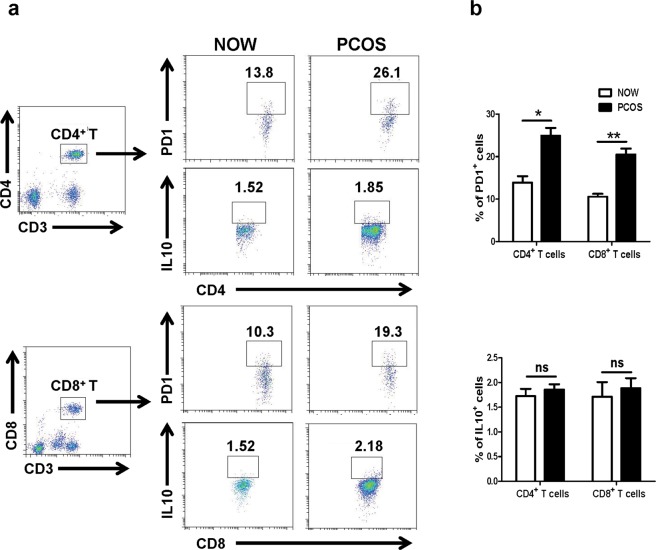
PD-1 and IL-10 expression on CD4^+^ and CD8^+^ T cells. Single cell suspensions were isolated from follicular fluid of NOW (n = 20) and infertile patients with PCOS (n = 20). CD3^+^CD4^+^ cells and CD3^+^CD8^+^ cells were first gated, and the expression of PD-1 on CD4^+^ and CD8^+^ T cells was detected using cell surface staining, while IL-10 expression was examined using intracellular cytokine staining, as previously described in the “Materials and methods” section. A representative result is shown (**a**). Average expressions of PD-1 and IL-10 on CD4^+^ and CD8^+^ T cells were calculated from the FACS data (**b**). *P < 0.05, compared with control group.

To examine the effect of PD-1 and IL-10 engagement on CD4^+^ and CD8^+^ T cell activation, cells from the follicular fluid of the patients with PCOS and the patients with normal ovulation were isolated. The expression of PD-1 was assayed by cell surface staining, while intracellular staining was used to detect IL-10 after stimulation with PMA plus ionomycin. As shown in Fig. 4a,b, the expression of PD-1 on CD4^+^ T cells in the PCOS group with infertility was significantly higher than that in the control group (13.80% ± 3.18% vs. 26.13% ± 3.31%, *P* < 0.05; 10.31% ± 2.34% vs. 19.30% ± 2.50%, *P* < 0.01). The percentages of IL-10-expressed CD4^+^ and CD8^+^ T cells in the PCOS group with infertility were slightly increased compared with the control group; however, there were no significant differences between the two groups (*P* > 0.05).

**Figure 5:**
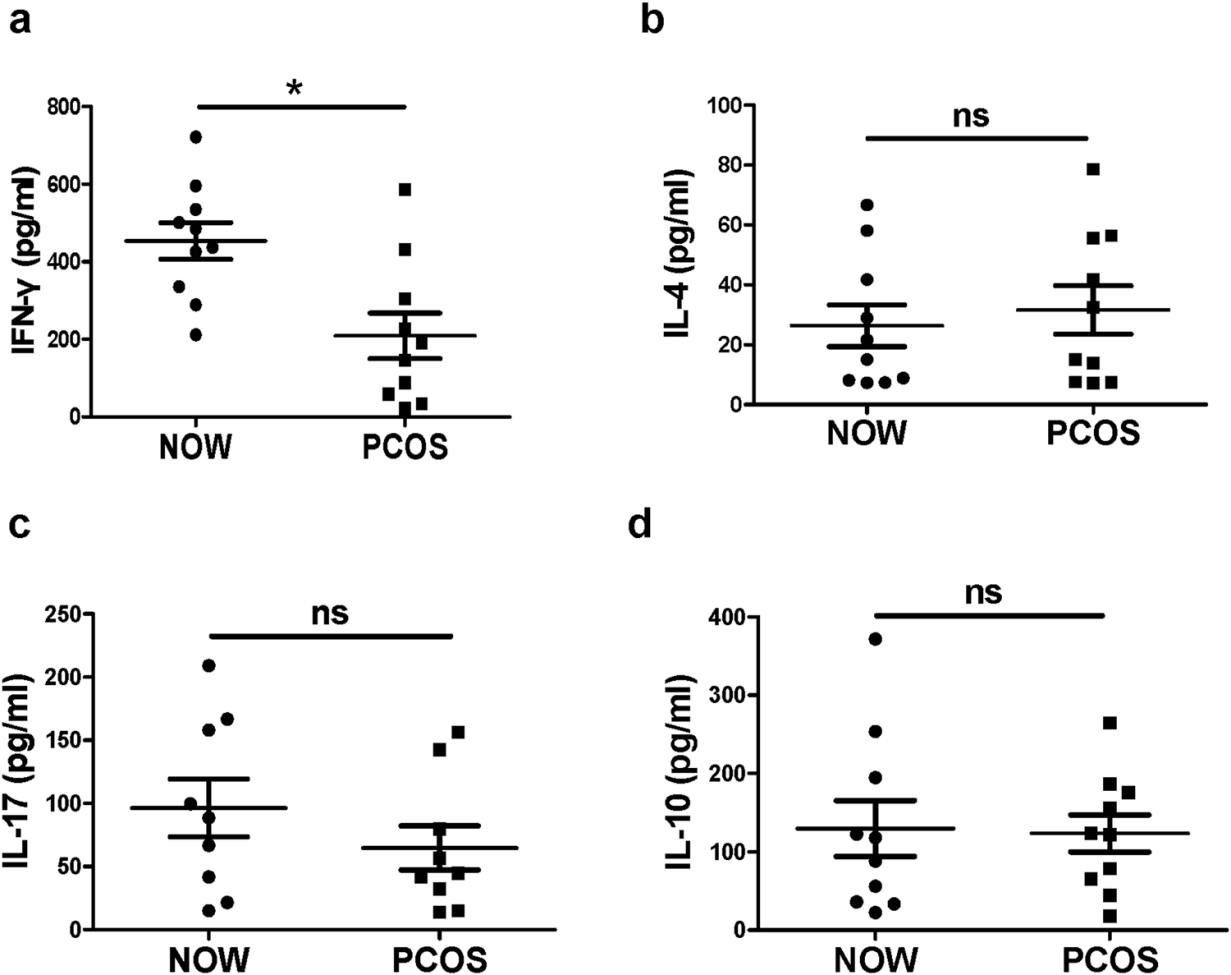
Cytokine expression profiles on CD4^+^ and CD8^+^ T cells by cytometric bead array kit (CBA). (**a**–**c**) Cells from follicular fluid of infertile patients with PCOS (n = 10) or NOW (n = 10) were stimulated with 20 ng/ml PMA and 1 μg/ml ionomycin and incubated for 48 h. The levels of IFN**-**γ (**a**), IL-4 (**b**), IL-17A (**c**) and IL-10 (**d**), all measured with cytometric bead array kit (CBA). *P < 0.05, compared with the control group.

We further confirmed the presence of intracellular cytokines using a cytometric bead array (CBA). Cells from the follicular fluid of the patients with PCOS or NOW were stimulated with 20 ng/ml PMA and 1 μg/ml ionomycin and incubated for 48 h. The levels of the cytokines IFN-γ, IL-10, IL-4 and IL-17A were analyzed in the cell culture supernatants by CBA. The results indicated that the level of IFN-γ was significantly reduced (423.6 ± 61.3 vs. 208.9 ± 53.5, pg/ml, *P* < 0.05) (Fig. 5a), while the levels of IL-4, IL-17A and IL-10 did not differ between the groups (*P* > 0.05) (Fig. 5b–d).

### Correlations between the PD-1 expression on CD4^+^ or CD8^+^ T cells in FF and serum E2 level, the IFN-γ expression in FF CD4^+^ or CD8^+^ T cells in infertile patients with PCOS

**Figure 6:**
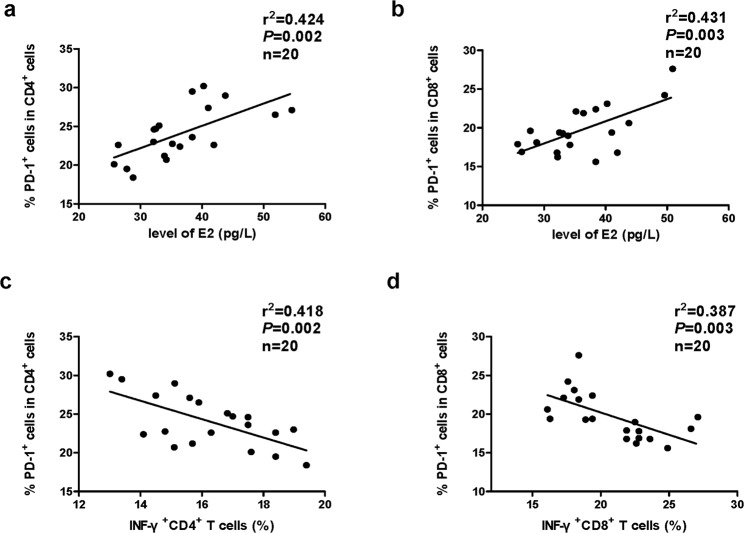
Correlations between the PD-1 expression on CD4^+^ or CD8^+^ T cells and the serum E2 level or IFN-γ expression on CD4^+^ or CD8^+^ T cells in infertile patients with PCOS. The PD-1 expression on CD4^+^ or CD8^+^ T cells was positively correlated with the E2 levels in serum (**a**,**b**) and reversely correlated with the IFN-γ expression on CD4^+^ or CD8^+^ T cells in infertile patients with PCOS (n = 20) (**c**,**d**). Pearson’s correlation test was used.

While the importance of T cells in the immune response has been demonstrated, a potential correlation of T cell exhaust with the ovarian response to gonadotropin stimulation is unknown. Our results showed that the PD-1 expression on CD4^+^ or CD8^+^ T cells, which reflects T cell exhaust, positively correlated with the serum E2 level in the infertile patients with PCOS (r^2^ = 0.424, *P* < 0.05 and r^2^ = 0.431, *P* < 0.05, respectively, Fig. 6a,b). The results further indicate that the exhaustion of T cells might be related to the development of oocytes and ovulation. Interestingly, inverse correlations between the expressions of PD-1 and IFN-γ in the FF CD4^+^ and CD8^+^ T cells were found in the infertile patients with PCOS (r^2^ = 0.418, *P* < 0.05 and r^2^ = 0.387, *P* < 0.05, respectively, Fig. 6c,d). These results indicated that the secretion of IFN-γ in T cells in PCOS patients with infertility may be suppressed by increased expression of PD-1.

## Discussion

Our results showed a significant reduction in the percentages of total CD3^+^ T cells and CD8^+^ T cells in the follicular fluid of the PCOS group with infertility compared with the control group (*P* < 0.05, Fig. 1). Consistent with previous reports, there were no disturbances in the percentages of CD4^+^ T cells in PCOS patients with infertility^31^. Although the percentage of CD4^+^ T cells did not change in PCOS patients with infertility, both CD4^+^ and CD8^+^ T cells expressed significantly lower levels of CD69 in the PCOS group (*P* < 0.01, Fig. 2). CD69 is the earliest molecule expressed on the cell surface of lymphocytes after activation^32^. These results further confirmed the existence of CD4^+^ and CD8^+^ T cell responses in PCOS patients with infertility, and the dysfunction of T cells might be associated with the pathogenesis of PCOS.

Recent studies have shown that several cytokines, including IFN-γ, TNF-α, IL-2, IL-4, IL-5, and IL-10, were produced by immunocompetent cells in the blood from PCOS with infertility *in vitro*, which might be involved in chronic inflammation^17^. Moreover, previous reports used a Cytometric Bead Array kit (CBA) to detect the production of these cytokines in cell culture supernatants. Our study is the first study to enrich lymphocytes from the follicular fluid and analyze the expression of IFN-γ, IL-4, IL17A, and IL10 in lymphocyte subsets of PCOS patients with infertility using flow cytometry.

IFN-γ has the ability to ‘interfere’ with the replication of virus in infected cells. Other effects of IFN-γ include the activation of macrophages, enhancing the activity of natural killer cells, synergy with cytokines and facilitating antibody production by cells^33^. IFN-γ-induced chemokines and their receptors play important roles in the pathogenesis of autoimmune endocrine diseases^34^. However, whether IFN-γ is involved in the pathogenesis of PCOS is not clear. Consistent with previous reports^17^, our results showed that untreated PCOS patients with infertility demonstrated significantly decreased expression of IFN-γ compared to women with normal ovulation (*P* < 0.05, Fig. 3a,b). Thus, the disordered levels of T cells and IFN-γ observed might cause the alteration of the mechanisms that regulate the expression of proteolytic enzymes, including collagenase and elastase, which can digest extracellular matrix proteins and thereby lead to follicular rupture and ovulation. As established, IL-4 can decrease the production of Th1 cells. However, our results showed that IL-4 could be rarely produced by CD4^+^ and CD8^+^ T cells in infertile patients with and without PCOS, and no significant difference was identified in the expression of IL-4 between the two groups (*P* > 0.05, Fig. 3a,b). Consistent with previous reports^35^, these results show that IL-4 might not be involved in the pathogenesis of PCOS.

Recent studies have shown that IL-17A is a major pro-inflammatory cytokine, which is associated with the interaction between PCOS and gingival inflammation^36^. However, in previous reports, ELISA was used to detect the production of IL-17A in gingival crevicular fluid (GCF), saliva, or serum. Our study is the first study to enrich lymphocytes from follicular fluid and analyze the IL-17A expression in lymphocyte subsets via flow cytometry analysis in PCOS patients in real time. Moreover, we found that IL-17A could be produced by CD4^+^ and CD8^+^ T cells in patients with and without PCOS; however, there was no significant difference in the expression of IL-17A between the two groups (*P* > 0.05, Fig. 3a,b). The results show that IL-17A might not be involved in the pathogenesis of PCOS.

PD-1 is crucial in mediating immune tolerance, infection, and cancer immunity^37^. As an inducible receptor, it has been reported to be expressed on peripheral T lymphocytes following activation. PD-1 inhibits antiviral T cell responses via the interaction with two ligands, PD-L1 and PD-L2^37,38^. As shown in Fig. 4a,b, our results indicated that the expression of PD-1 in FF CD4^+^ or CD8^+^ T cells from the PCOS group with infertility was significantly higher than that from the control group (*P* < 0.05). Furthermore, patients with PCOS showed an inverse correlation between the expression of PD-1 and IFN-γ in FF CD4^+^ or CD8^+^ T cells (*P* < 0.05, Fig. 6c,d). These findings indicated that the survival and activation of T cells in PCOS patients with infertility might be suppressed by increased expression of PD-1. IL-10 markedly inhibits the functions of monocytes-macrophages, such as antigen presentation^39^. As a potent inhibitory molecule, IL-10 restrains the lytic activity of CD4^+^ and CD8^+^ T cells^40^. Moreover, IL-10 could be detected in both infertile patients with and without PCOS; however, no difference was observed in the percentage of IL-10^+^CD4^+^ or IL-10^+^CD8^+^ cells between the two groups (P > 0.05, Fig. 4a,b).

Follicular granulosa cells can produce a supraphysiological level of serum E2 during controlled ovarian hyperstimulation, which is associated with the development of multiple ovarian follicles, and the level of serum E2 correlated with the maturity and quality of ovarian follicles^41^. Interestingly, we found that the serum E2 level positively correlated with the expression of PD-1 in FF CD4^+^ or CD8^+^ T cells. Furthermore, an inverse correlation between the expression of PD-1 and IFN-γ in FF CD4^+^ or CD8^+^ T cells was found in infertile patients with PCOS (*P* < 0.05, Fig. 6). The results showed that abnormal activation of T cells and cytokine production might lead to the abnormal oocyte development observed in PCOS patients. Recent studies have shown that metabolic dysbalance plays a key role in PCOS pathogenesis, and inositol supplementation could reduce the amount of gonadotropins and the length of ovarian stimulation in women undergoing IVF^42–44^. Consequently, we propose that the correction of T cell dysfunction may re-address hormonal and clinical parameters to restore homeostasis.

PCOS is the most prevalent endocrinopathy of reproductive-aged women. However, infertility occurs in about 10–20% of patients with PCOS^45,46^. In our study, there is a potential selection bias since the study only included infertile patients with or without PCOS, which might be a surrogate for the severity/chronicity of the disease. To get more reliable results, the study should include a random sample of all PCOS patients. However, the department we work in is the reproductive medical center, it is difficult to obtain clinical samples from PCOS patients with fertility. Furthermore, even in PCOS patients, follicular fluid samples will not be taken during examination and treatment if the pregnancy is normal. Thus, we only focused on PCOS in infertile patients and limited our findings to infertile patients with PCOS in this study.

In summary, this report found that increased expression of PD-1 and significantly decreased expression of IFN-γ were detected in CD4^+^ T and CD8^+^ T cells in infertile patients with PCOS (P < 0.05). We speculate that the higher expression of PD-1 in CD4^+^ T and CD8^+^ T cells in the FF in PCOS patients with infertility probably cannot induce T cell activation or recruitment, which, in turn, leads to the failure of dominant follicle selection and development. It is concluded that the dysfunction of T cells, which may be an immunological feature, might participate in the immune pathogenesis in the ovary of PCOS patients with infertility. These results suggest that chronic inflammation may be one of the underlying mechanisms for the pathogenesis of PCOS.
